# Supplementary material for: Reconciling Mining with the Conservation of Cave Biodiversity: A Quantitative Baseline to Help Establish Conservation Priorities
Source: PLoS One. 2016 Dec 20;11(12):e0168348. doi: 10.1371/journal.pone.0168348 (PMC5173368; doi:10.1371/journal.pone.0168348)
Supplement: S1 Dataset — (ZIP) [file pone.0168348.s002.zip › Taxa/Serra Norte/SN_2013/N4E/Lista N4E-11.pdf]

CAVIDADE N4E-0011

Classe	Ordem	Fam/Outros	Gên/Outros	Espécie	S-U	U-S	Zon.
Annelida	Oligochaeta			jovem		1	P
Arachnida	Acari	(Oribatida)		jovem	X		
Arachnida	Acari	(Parasitengonina)		sp.9		X	P
Arachnida	Acari	Laelapidae		sp.4		X	P
Arachnida	Acari	Trombiculidae		sp.39	X	X	P
Arachnida	Amblypygi	Phrynidae	*Heterophrynus*	*longicornis*	X		
Arachnida	Araneae	Araneidae		jovem	X	X	P
Arachnida	Araneae	Ctenidae		jovem	1		
Arachnida	Araneae	Drymusidae	*Drymusa*	*spelunca*		X	P
Arachnida	Araneae	Mysmenidae		jovem	X	X	E
Arachnida	Araneae	Ochyroceratidae	*Ochyrocera*	sp.	X		
Arachnida	Araneae	Ochyroceratidae	*Ochyrocera*	sp.1		X	P
Arachnida	Araneae	Ochyroceratidae	*Ochyrocera*	sp.3		X	P
Arachnida	Araneae	Ochyroceratidae	*Speocera*	sp.1	X	X	P
Arachnida	Araneae	Pholcidae	*Mesabolivar*	sp.	X	X	
Arachnida	Araneae	Scytodidae	*Scytodes*	sp.	X		
Arachnida	Araneae	Theridiidae	*Theridion*	sp.1		X	E
Arachnida	Araneae	Theridiidae	*Thymoites*	sp.	X		
Arachnida	Araneae	Theridiosomatidae	*Plato*	sp.1	X	X	E/P
Arachnida	Opiliones	Escadabiidae		sp.2		X	P
Arachnida	Opiliones	Stygnidae	*Protimesius*	*gracilis*	X		
Arachnida	Palpigradi	Eukoeneniidae	*Allokoenenia*	sp.1		X	P
Arachnida	Pseudoscorpiones	Chernetidae	*Spelaeochernes*	sp.1	X	X	E/P
Arachnida	Pseudoscorpiones	Chthoniidae	*Pseudochthonius*	sp.1		X	E/P
Chilopoda	Geophilomorpha	Geophilidae		sp.1		1	P
Chilopoda	Scolopendromorpha	Cryptopidae	*Cryptops*	jovem		1	P
Chilopoda	Scolopendromorpha	Scolopocryptopidae	*Newportia*	sp.	X		
Chilopoda	Scutigeromorpha	Pselliodidae	*Sphendononema*	*guildingii*	X		
Diplopoda	Polydesmida	Chelodesmidae		jovem	X	1	P
Diplopoda	Polydesmida	Fuhrmannodesmidae		jovem		X	P
Diplopoda	Polydesmida	Pyrgodesmidae		sp.1	X	X	P
Diplopoda	Spirostreptida	Pseudonannolenidae	*Pseudonannolene*	jovem		1	P
Entognatha	Collembola	Isotomidae		sp.1		X	P
Entognatha	Collembola	Paronellidae		sp.1		X	E
Entognatha	Collembola	Paronellidae		sp.4		X	P
Entognatha	Collembola	Sminthuroidea		sp.2		X	P
Entognatha	Collembola			sp.	X		
Entognatha	Diplura	Campodeidae		sp.1	X	X	E/P
Entognatha	Diplura	Projapygidae		sp.1		X	P
Insecta	Blattodea	Polyphagidae		jovem	1	2	E
Insecta	Coleoptera	Curculionidae		sp.1	X		
Insecta	Coleoptera	Staphylinidae		sp.33	X		
Insecta	Coleoptera	Staphylinidae		sp.76	X	X	P
Insecta	Diptera	Chironomidae		sp.		X	E
Insecta	Diptera	Psychodidae	*Sciopemyia*	*sordellii*	X	X	
Insecta	Diptera	Tipulidae		sp.	X		
Insecta	Heteroptera	Cydnidae		sp.	X		
Insecta	Heteroptera	Reduviidae	Emesinae	sp.		1	E
Insecta	Heteroptera	Reduviidae	*Zelurus*	sp.	X	1	E
Insecta	Homoptera	Cixiidae		jovem	X	X	E/P
Insecta	Hymenoptera	Formicidae	*Camponotus*	sp.1		X	P
Insecta	Hymenoptera	Formicidae	*Crematogaster*	sp.2		X	P
Insecta	Hymenoptera	Formicidae	*Dolichoderus*	aff. *bispinosus*		X	E
Insecta	Hymenoptera	Formicidae	*Pachycondyla*	sp.2		X	E
Insecta	Hymenoptera	Formicidae	*Pheidole*	sp.	X		

Insecta	Isoptera	Termitidae	*Nasutitermes*	sp.	X	X	E
Insecta	Isoptera	Termitidae	*Termes*	sp.		X	P
Insecta	Lepidoptera	Noctuoidea		sp.5		1	E
Insecta	Lepidoptera	Tineoidea		jovem	X		
Insecta	Plecoptera	Gripopterygidae		jovem		1	P
Insecta	Psocoptera			jovem	X	X	E/P
Gastropoda	Pulmonata	Subulinidae	*Obeliscus*	sp.	X		
Gastropoda	Pulmonata	Systrophiidae	*Happia*	sp.	X		
Malacostraca	Isopoda	Armadillidae		sp.1		X	P
Malacostraca	Isopoda	Scleropactidae	*Circoniscus*	*buckupi*	X		
Symphyla		Scutigerellidae		sp.		X	P
Amphibia	Anura			sp.	X	2	P
Mammalia	Chiroptera	Emballonuridae	*Peropteryx*	sp.		2	P
Mammalia	Chiroptera	Phyllostomidae	*Glossophaga*	*soricina*		1	P
Mammalia	Chiroptera			sp.	30		P
Mammalia	Rodentia	Cricetidae	*Rhipidomys*	sp.	4		P
